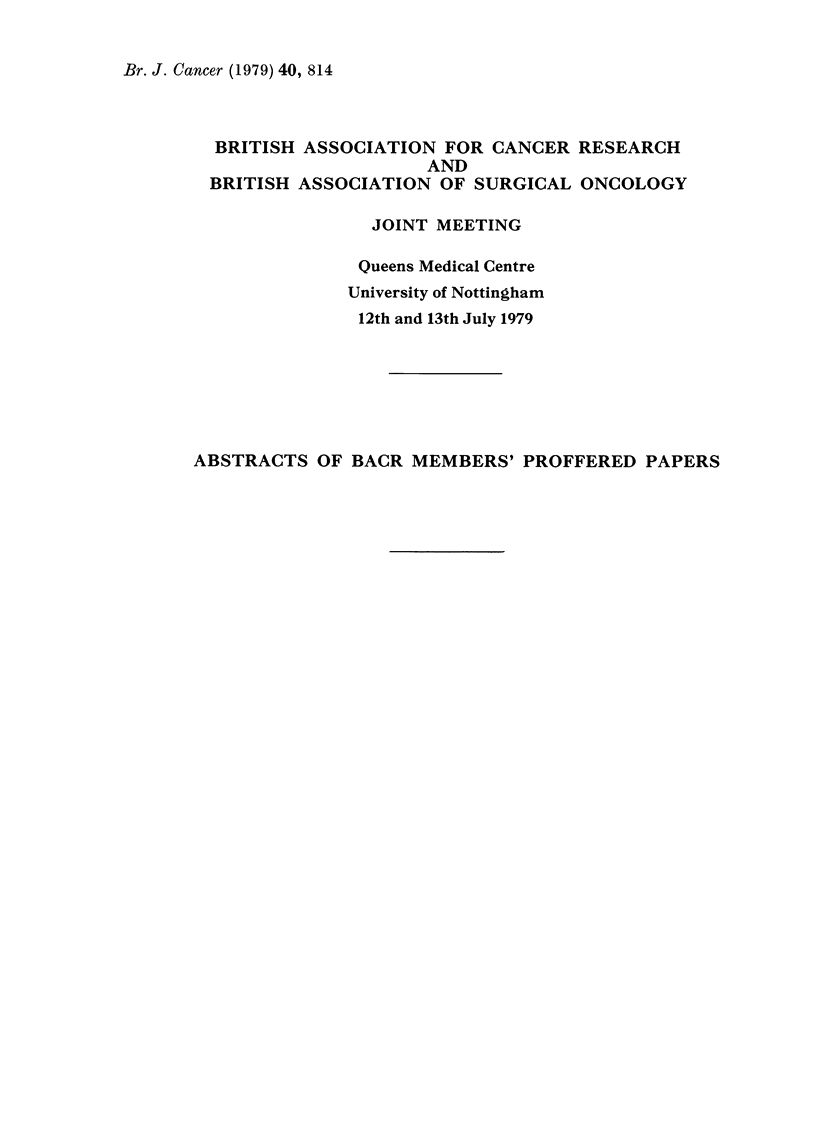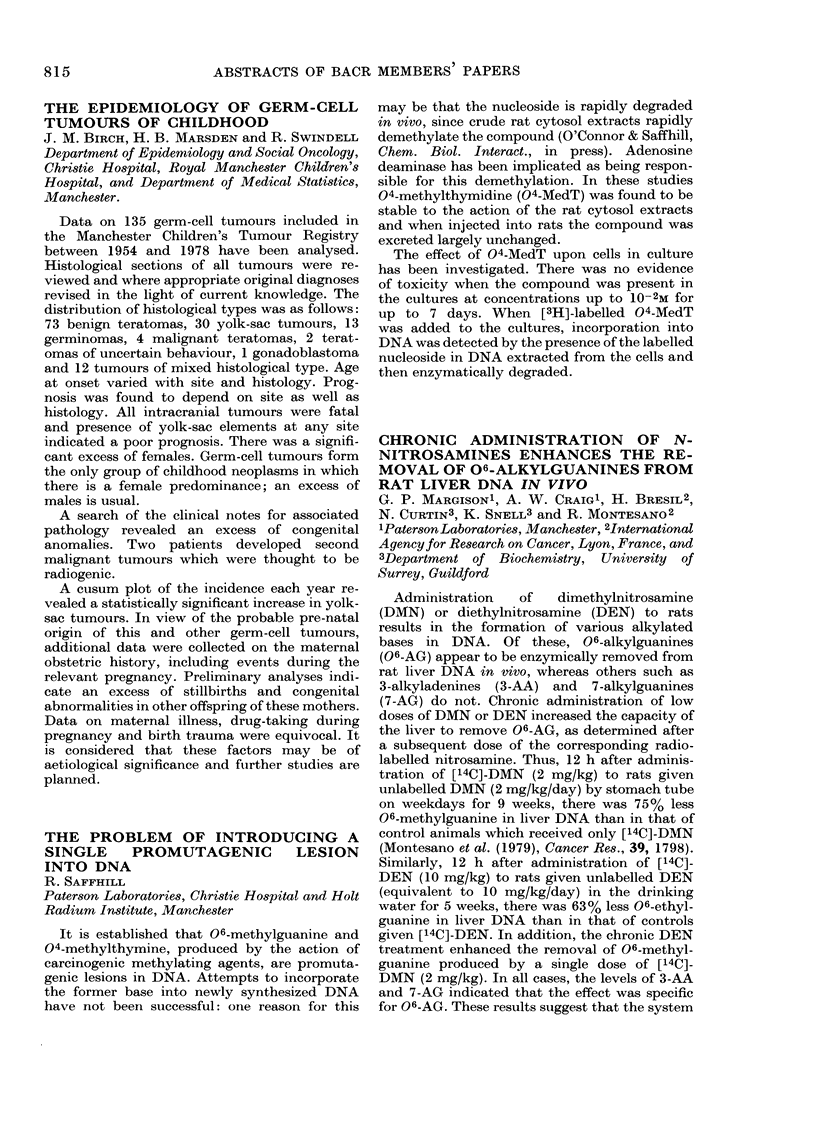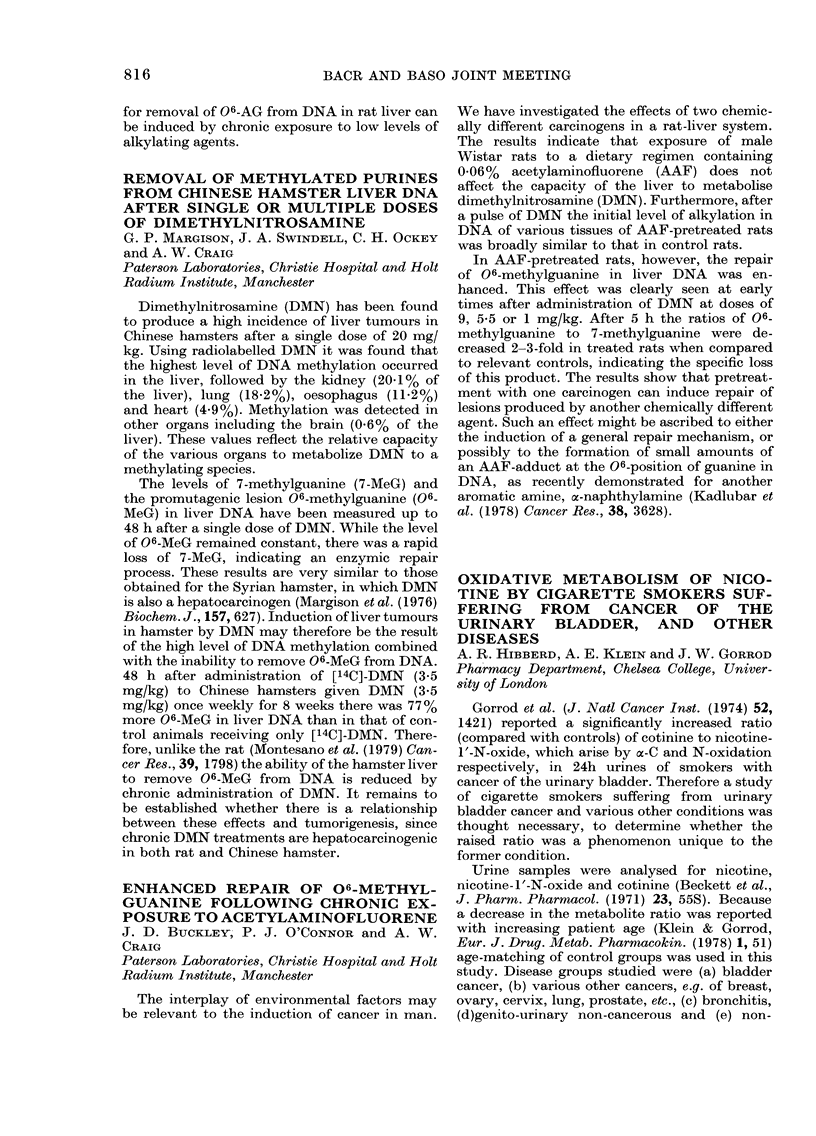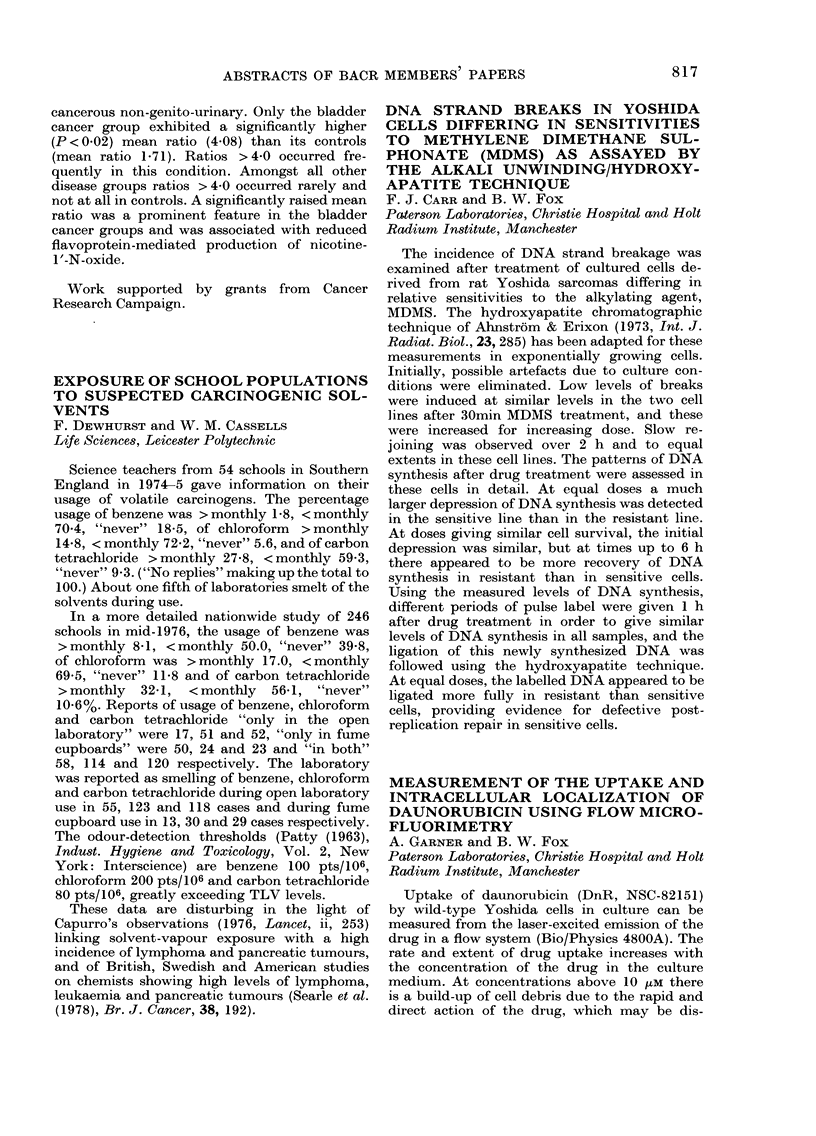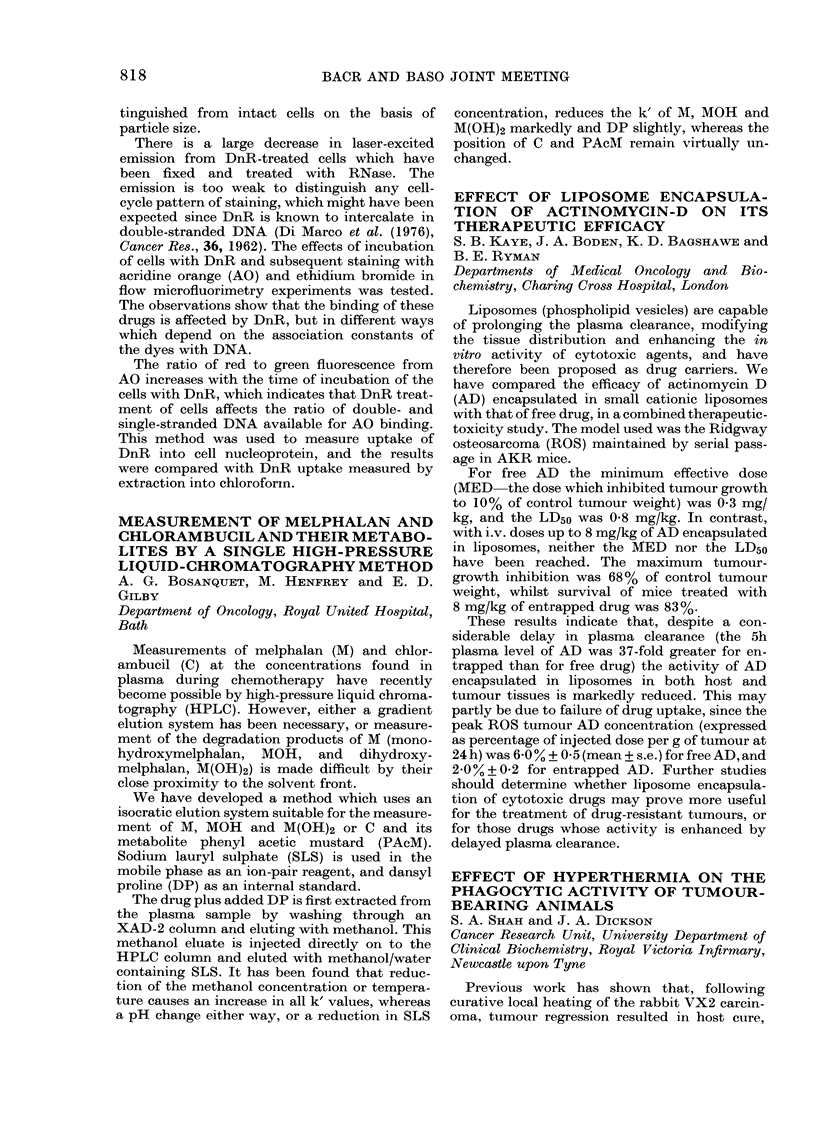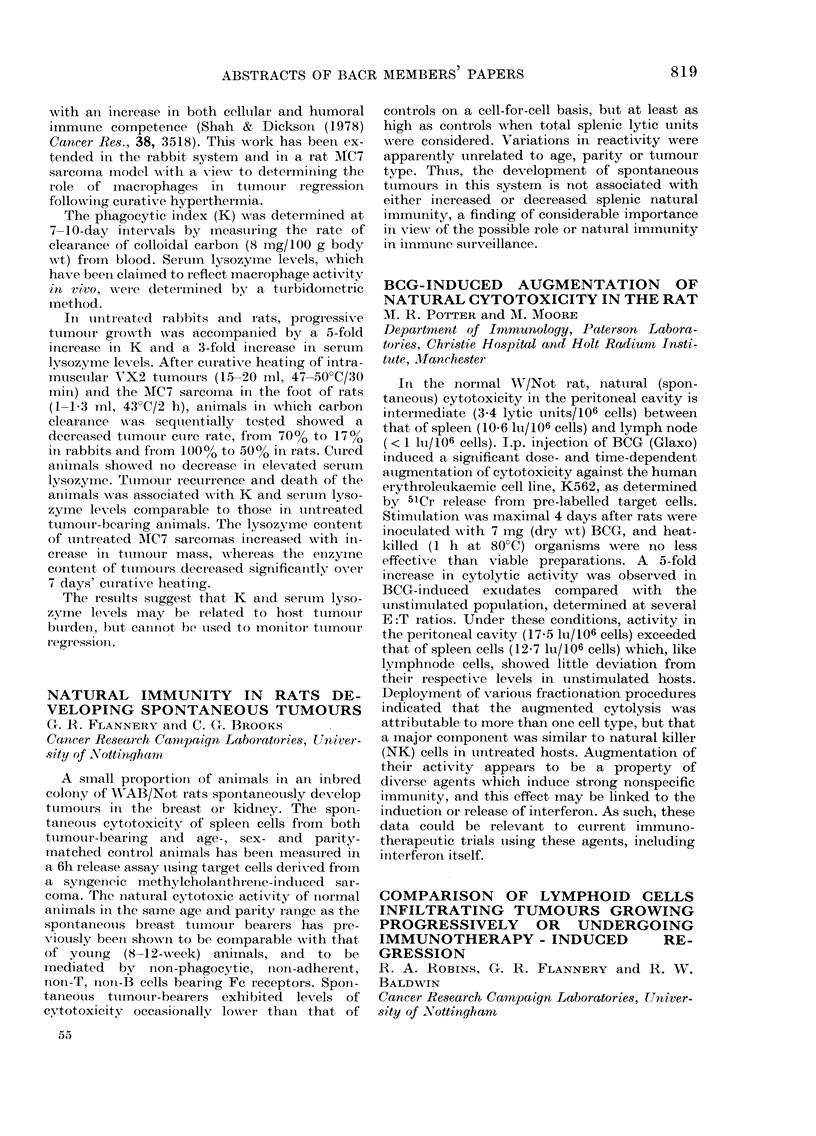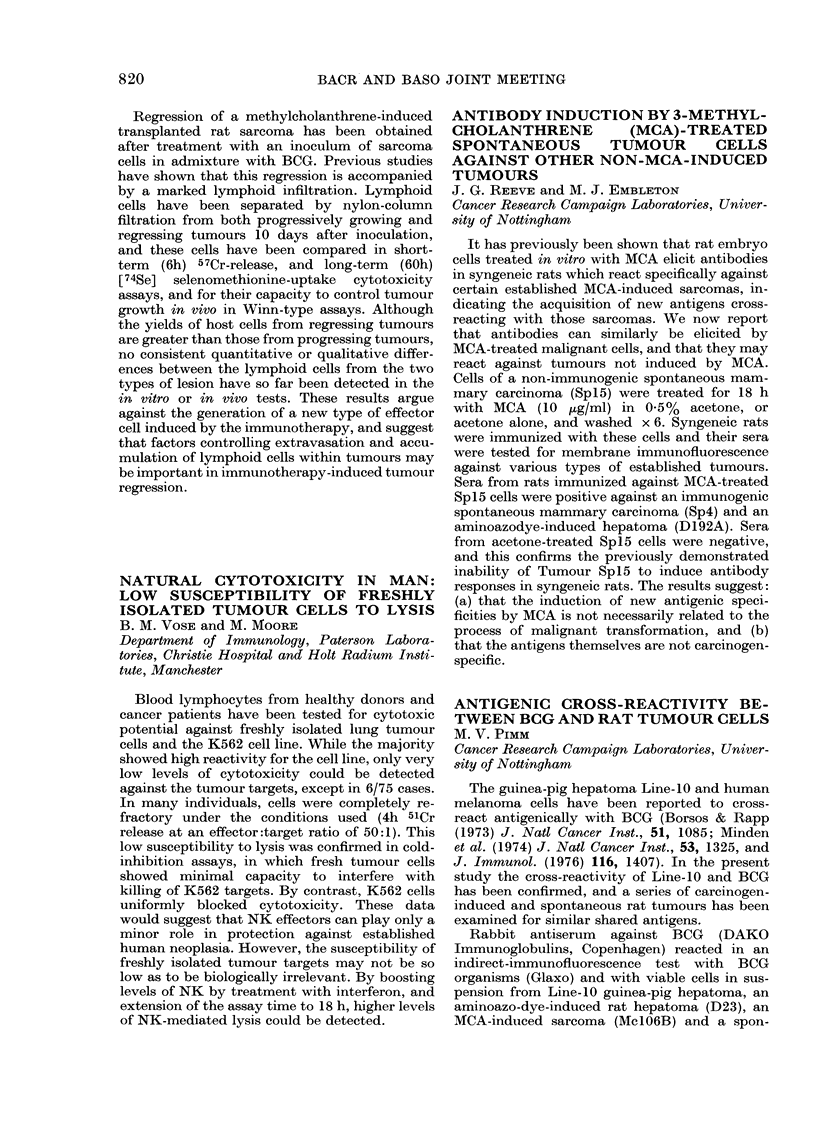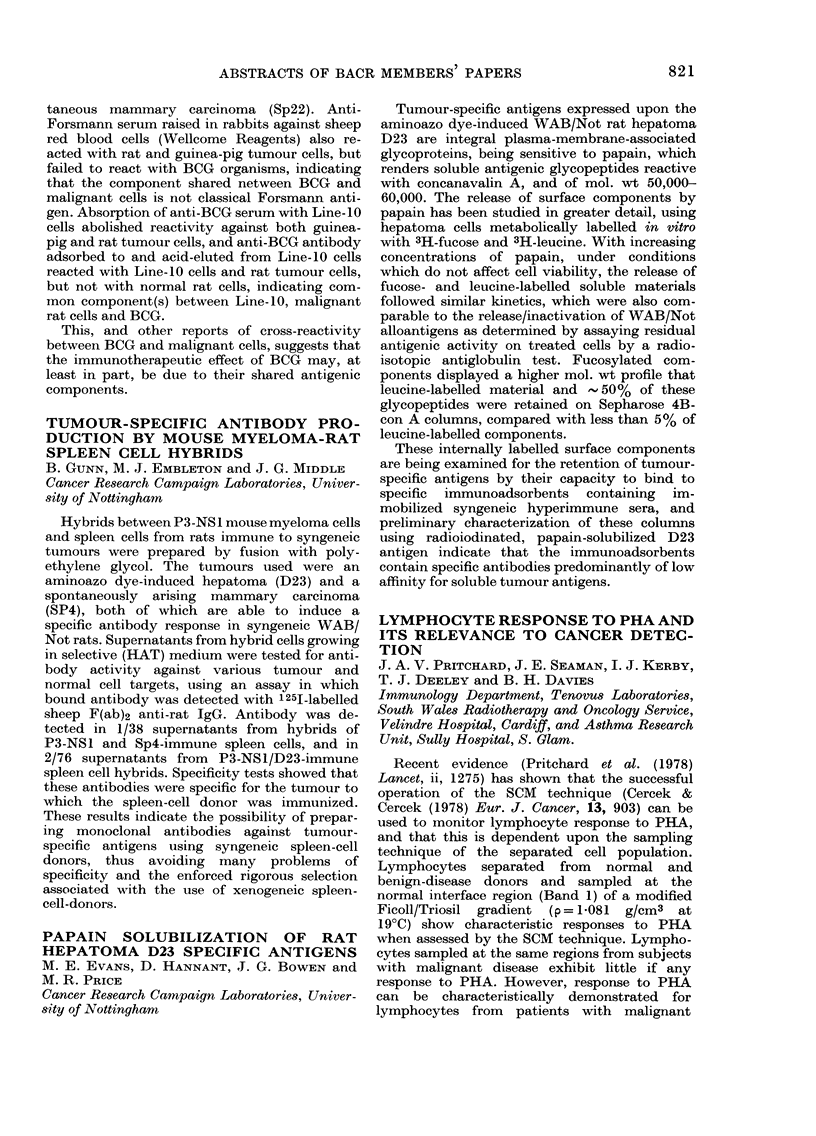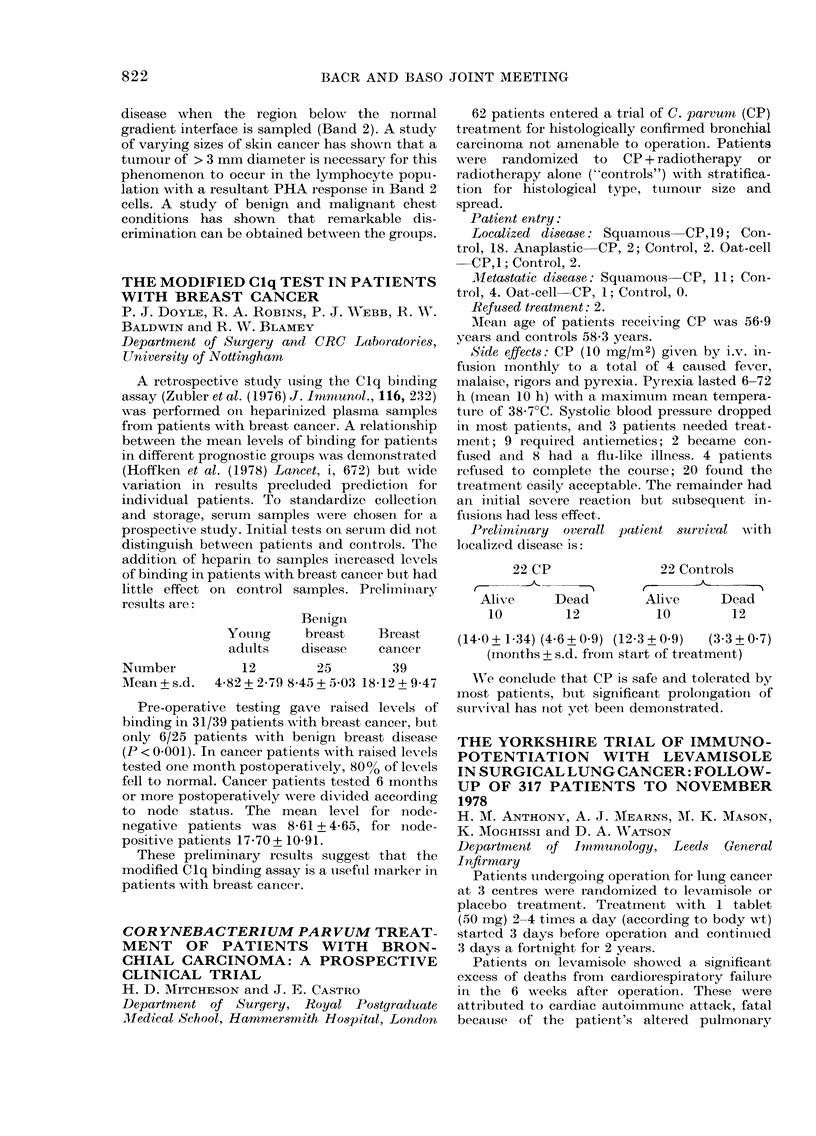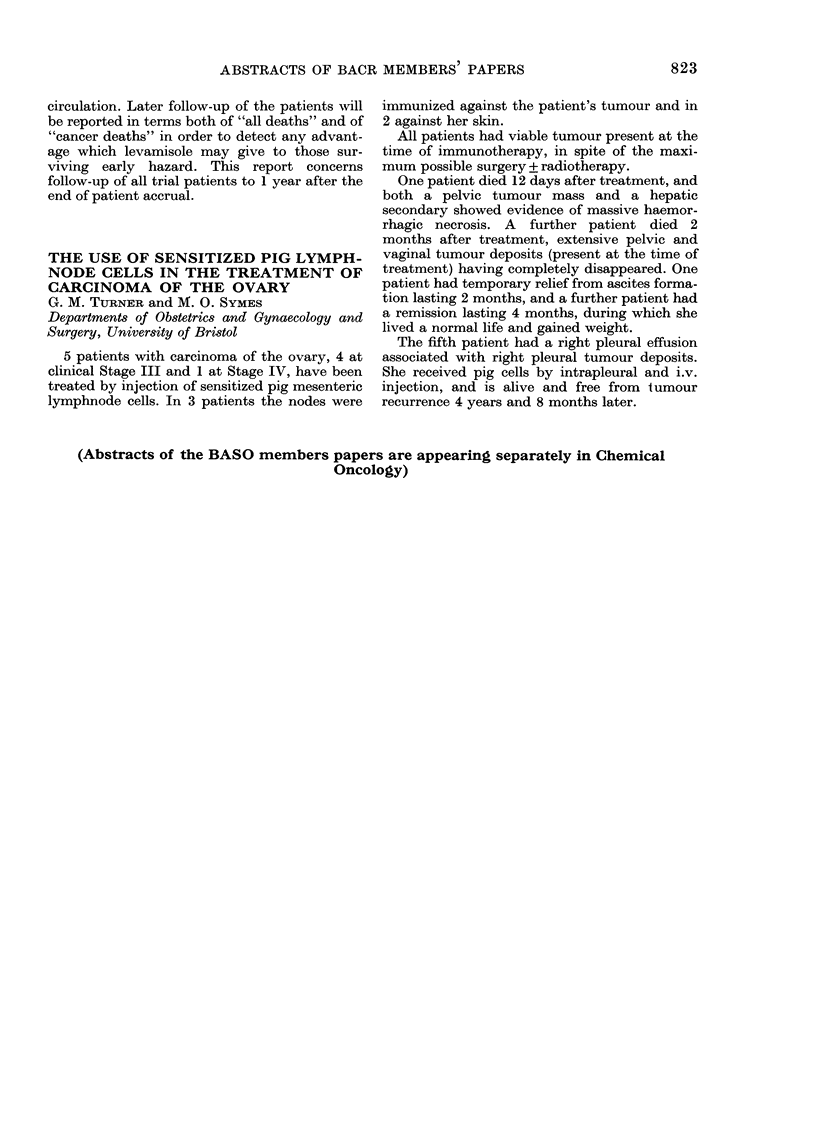# BACR Abstracts

**Published:** 1979-11

**Authors:** 


*Br. J. Cancer* (1979) **40**, 814

### BRITISH ASSOCIATION FOR CANCER RESEARCH
### AND
### BRITISH ASSOCIATION OF SURGICAL ONCOLOGY

### JOINT MEETING

**Queens Medical Centre**

**University of Nottingham**

**12th and 13th July 1979**

---

### ABSTRACTS OF BACR MEMBERS' PROFFERED PAPERS

---

### THE EPIDEMIOLOGY OF GERM-CELL TUMOURS OF CHILDHOOD

J. M. BIRCH, H. B. MARSDEN and R. SWINDELL
*Department of Epidemiology and Social Oncology, Christie Hospital, Royal Manchester Children's Hospital, and Department of Medical Statistics, Manchester.*

Data on 135 germ-cell tumours included in the Manchester Children's Tumour Registry between 1954 and 1978 have been analysed. Histological sections of all tumours were reviewed and where appropriate original diagnoses revised in the light of current knowledge. The distribution of histological types was as follows: 73 benign teratomas, 30 yolk-sac tumours, 13 germinomas, 4 malignant teratomas, 2 teratomas of uncertain behaviour, 1 gonadoblastoma and 12 tumours of mixed histological type. Age at onset varied with site and histology. Prognosis was found to depend on site as well as histology. All intracranial tumours were fatal and presence of yolk-sac elements at any site indicated a poor prognosis. There was a significant excess of females. Germ-cell tumours form the only group of childhood neoplasms in which there is a female predominance; an excess of males is usual.

A search of the clinical notes for associated pathology revealed an excess of congenital anomalies. Two patients developed second malignant tumours which were thought to be radiogenic.

A cusum plot of the incidence each year revealed a statistically significant increase in yolk-sac tumours. In view of the probable pre-natal origin of this and other germ-cell tumours, additional data were collected on the maternal obstetric history, including events during the relevant pregnancy. Preliminary analyses indicate an excess of stillbirths and congenital abnormalities in other offspring of these mothers. Data on maternal illness, drug-taking during pregnancy and birth trauma were equivocal. It is considered that these factors may be of aetiological significance and further studies are planned.

### THE PROBLEM OF INTRODUCING A SINGLE PROMUTAGENIC LESION INTO DNA

R. SAFFHILL
*Paterson Laboratories, Christie Hospital and Holt Radium Institute, Manchester*

It is established that $O^6$-methylguanine and $O^4$-methylthymine, produced by the action of carcinogenic methylating agents, are promutagenic lesions in DNA. Attempts to incorporate the former base into newly synthesized DNA have not been successful: one reason for this may be that the nucleoside is rapidly degraded *in vivo*, since crude rat cytosol extracts rapidly demethylate the compound (O'Connor & Saffhill, *Chem. Biol. Interact.*, in press). Adenosine deaminase has been implicated as being responsible for this demethylation. In these studies $O^4$-methylthymidine ($O^4$-MedT) was found to be stable to the action of the rat cytosol extracts and when injected into rats the compound was excreted largely unchanged.

The effect of $O^4$-MedT upon cells in culture has been investigated. There was no evidence of toxicity when the compound was present in the cultures at concentrations up to $10^{-2}$M for up to 7 days. When [$^3$H]-labelled $O^4$-MedT was added to the cultures, incorporation into DNA was detected by the presence of the labelled nucleoside in DNA extracted from the cells and then enzymatically degraded.

### CHRONIC ADMINISTRATION OF *N*-NITROSAMINES ENHANCES THE REMOVAL OF O$^6$-ALKYLGUANINES FROM RAT LIVER DNA *IN VIVO*

G. P. MARGISON[1], A. W. CRAIG[1], H. BRESIL[2], N. CURTIN[3], K. SNELL[3] and R. MONTESANO[2]
[1]*Paterson Laboratories, Manchester,* [2]*International Agency for Research on Cancer, Lyon, France, and* [3]*Department of Biochemistry, University of Surrey, Guildford*

Administration of dimethylnitrosamine (DMN) or diethylnitrosamine (DEN) to rats results in the formation of various alkylated bases in DNA. Of these, $O^6$-alkylguanines ($O^6$-AG) appear to be enzymically removed from rat liver DNA *in vivo*, whereas others such as 3-alkyladenines (3-AA) and 7-alkylguanines (7-AG) do not. Chronic administration of low doses of DMN or DEN increased the capacity of the liver to remove $O^6$-AG, as determined after a subsequent dose of the corresponding radiolabelled nitrosamine. Thus, 12 h after administration of [$^{14}$C]-DMN (2 mg/kg) to rats given unlabelled DMN (2 mg/kg/day) by stomach tube on weekdays for 9 weeks, there was 75% less $O^6$-methylguanine in liver DNA than in that of control animals which received only [$^{14}$C]-DMN (Montesano *et al.* (1979), *Cancer Res.*, **39**, 1798). Similarly, 12 h after administration of [$^{14}$C]-DEN (10 mg/kg) to rats given unlabelled DEN (equivalent to 10 mg/kg/day) in the drinking water for 5 weeks, there was 63% less $O^6$-ethylguanine in liver DNA than in that of controls given [$^{14}$C]-DEN. In addition, the chronic DEN treatment enhanced the removal of $O^6$-methylguanine produced by a single dose of [$^{14}$C]-DMN (2 mg/kg). In all cases, the levels of 3-AA and 7-AG indicated that the effect was specific for $O^6$-AG. These results suggest that the system for removal of $O^6$-AG from DNA in rat liver can be induced by chronic exposure to low levels of alkylating agents.

### REMOVAL OF METHYLATED PURINES FROM CHINESE HAMSTER LIVER DNA AFTER SINGLE OR MULTIPLE DOSES OF DIMETHYLNITROSAMINE

G. P. MARGISON, J. A. SWINDELL, C. H. OCKEY and A. W. CRAIG
*Paterson Laboratories, Christie Hospital and Holt Radium Institute, Manchester*

Dimethylnitrosamine (DMN) has been found to produce a high incidence of liver tumours in Chinese hamsters after a single dose of 20 mg/kg. Using radiolabelled DMN it was found that the highest level of DNA methylation occurred in the liver, followed by the kidney (20·1% of the liver), lung (18·2%), oesophagus (11·2%) and heart (4·9%). Methylation was detected in other organs including the brain (0·6% of the liver). These values reflect the relative capacity of the various organs to metabolize DMN to a methylating species.

The levels of 7-methylguanine (7-MeG) and the promutagenic lesion $O^6$-methylguanine ($O^6$-MeG) in liver DNA have been measured up to 48 h after a single dose of DMN. While the level of $O^6$-MeG remained constant, there was a rapid loss of 7-MeG, indicating an enzymic repair process. These results are very similar to those obtained for the Syrian hamster, in which DMN is also a hepatocarcinogen (Margison *et al.* (1976) *Biochem. J.*, **157,** 627). Induction of liver tumours in hamster by DMN may therefore be the result of the high level of DNA methylation combined with the inability to remove $O^6$-MeG from DNA. 48 h after administration of [$^{14}$C]-DMN (3·5 mg/kg) to Chinese hamsters given DMN (3·5 mg/kg) once weekly for 8 weeks there was 77% more $O^6$-MeG in liver DNA than in that of control animals receiving only [$^{14}$C]-DMN. Therefore, unlike the rat (Montesano *et al.* (1979) *Cancer Res.*, **39,** 1798) the ability of the hamster liver to remove $O^6$-MeG from DNA is reduced by chronic administration of DMN. It remains to be established whether there is a relationship between these effects and tumorigenesis, since chronic DMN treatments are hepatocarcinogenic in both rat and Chinese hamster.

### ENHANCED REPAIR OF $O^6$-METHYLGUANINE FOLLOWING CHRONIC EXPOSURE TO ACETYLAMINOFLUORENE

J. D. BUCKLEY, P. J. O'CONNOR and A. W. CRAIG
*Paterson Laboratories, Christie Hospital and Holt Radium Institute, Manchester*

The interplay of environmental factors may be relevant to the induction of cancer in man. We have investigated the effects of two chemically different carcinogens in a rat-liver system. The results indicate that exposure of male Wistar rats to a dietary regimen containing 0·06% acetylaminofluorene (AAF) does not affect the capacity of the liver to metabolise dimethylnitrosamine (DMN). Furthermore, after a pulse of DMN the initial level of alkylation in DNA of various tissues of AAF-pretreated rats was broadly similar to that in control rats.

In AAF-pretreated rats, however, the repair of $O^6$-methylguanine in liver DNA was enhanced. This effect was clearly seen at early times after administration of DMN at doses of 9, 5·5 or 1 mg/kg. After 5 h the ratios of $O^6$-methylguanine to 7-methylguanine were decreased 2–3-fold in treated rats when compared to relevant controls, indicating the specific loss of this product. The results show that pretreatment with one carcinogen can induce repair of lesions produced by another chemically different agent. Such an effect might be ascribed to either the induction of a general repair mechanism, or possibly to the formation of small amounts of an AAF-adduct at the $O^6$-position of guanine in DNA, as recently demonstrated for another aromatic amine, α-naphthylamine (Kadlubar *et al.* (1978) *Cancer Res.*, **38,** 3628).

### OXIDATIVE METABOLISM OF NICOTINE BY CIGARETTE SMOKERS SUFFERING FROM CANCER OF THE URINARY BLADDER, AND OTHER DISEASES

A. R. HIBBERD, A. E. KLEIN and J. W. GORROD
*Pharmacy Department, Chelsea College, University of London*

Gorrod *et al.* (*J. Natl Cancer Inst.* (1974) **52,** 1421) reported a significantly increased ratio (compared with controls) of cotinine to nicotine-1′-N-oxide, which arise by α-C and N-oxidation respectively, in 24h urines of smokers with cancer of the urinary bladder. Therefore a study of cigarette smokers suffering from urinary bladder cancer and various other conditions was thought necessary, to determine whether the raised ratio was a phenomenon unique to the former condition.

Urine samples were analysed for nicotine, nicotine-1′-N-oxide and cotinine (Beckett *et al.*, *J. Pharm. Pharmacol.* (1971) **23,** 55S). Because a decrease in the metabolite ratio was reported with increasing patient age (Klein & Gorrod, *Eur. J. Drug. Metab. Pharmacokin.* (1978) **1,** 51) age-matching of control groups was used in this study. Disease groups studied were (a) bladder cancer, (b) various other cancers, *e.g.* of breast, ovary, cervix, lung, prostate, *etc.*, (c) bronchitis, (d)genito-urinary non-cancerous and (e) noncancerous non-genito-urinary. Only the bladder cancer group exhibited a significantly higher ($P < 0{\cdot}02$) mean ratio (4·08) than its controls (mean ratio 1·71). Ratios $> 4{\cdot}0$ occurred frequently in this condition. Amongst all other disease groups ratios $> 4{\cdot}0$ occurred rarely and not at all in controls. A significantly raised mean ratio was a prominent feature in the bladder cancer groups and was associated with reduced flavoprotein-mediated production of nicotine-1′-N-oxide.

Work supported by grants from Cancer Research Campaign.

### EXPOSURE OF SCHOOL POPULATIONS TO SUSPECTED CARCINOGENIC SOLVENTS

F. DEWHURST and W. M. CASSELLS
*Life Sciences, Leicester Polytechnic*

Science teachers from 54 schools in Southern England in 1974–5 gave information on their usage of volatile carcinogens. The percentage usage of benzene was >monthly 1·8, <monthly 70·4, "never" 18·5, of chloroform >monthly 14·8, <monthly 72·2, "never" 5.6, and of carbon tetrachloride >monthly 27·8, <monthly 59·3, "never" 9·3. ("No replies" making up the total to 100.) About one fifth of laboratories smelt of the solvents during use.

In a more detailed nationwide study of 246 schools in mid-1976, the usage of benzene was >monthly 8·1, <monthly 50.0, "never" 39·8, of chloroform was >monthly 17.0, <monthly 69·5, "never" 11·8 and of carbon tetrachloride >monthly 32·1, <monthly 56·1, "never" 10·6%. Reports of usage of benzene, chloroform and carbon tetrachloride "only in the open laboratory" were 17, 51 and 52, "only in fume cupboards" were 50, 24 and 23 and "in both" 58, 114 and 120 respectively. The laboratory was reported as smelling of benzene, chloroform and carbon tetrachloride during open laboratory use in 55, 123 and 118 cases and during fume cupboard use in 13, 30 and 29 cases respectively. The odour-detection thresholds (Patty (1963), *Indust. Hygiene and Toxicology*, Vol. 2, New York: Interscience) are benzene 100 pts/$10^6$, chloroform 200 pts/$10^6$ and carbon tetrachloride 80 pts/$10^6$, greatly exceeding TLV levels.

These data are disturbing in the light of Capurro's observations (1976, *Lancet*, ii, 253) linking solvent-vapour exposure with a high incidence of lymphoma and pancreatic tumours, and of British, Swedish and American studies on chemists showing high levels of lymphoma, leukaemia and pancreatic tumours (Searle *et al.* (1978), *Br. J. Cancer*, **38,** 192).

### DNA STRAND BREAKS IN YOSHIDA CELLS DIFFERING IN SENSITIVITIES TO METHYLENE DIMETHANE SULPHONATE (MDMS) AS ASSAYED BY THE ALKALI UNWINDING/HYDROXYAPATITE TECHNIQUE

F. J. CARR and B. W. FOX
*Paterson Laboratories, Christie Hospital and Holt Radium Institute, Manchester*

The incidence of DNA strand breakage was examined after treatment of cultured cells derived from rat Yoshida sarcomas differing in relative sensitivities to the alkylating agent, MDMS. The hydroxyapatite chromatographic technique of Ahnström & Erixon (1973, *Int. J. Radiat. Biol.*, **23,** 285) has been adapted for these measurements in exponentially growing cells. Initially, possible artefacts due to culture conditions were eliminated. Low levels of breaks were induced at similar levels in the two cell lines after 30min MDMS treatment, and these were increased for increasing dose. Slow rejoining was observed over 2 h and to equal extents in these cell lines. The patterns of DNA synthesis after drug treatment were assessed in these cells in detail. At equal doses a much larger depression of DNA synthesis was detected in the sensitive line than in the resistant line. At doses giving similar cell survival, the initial depression was similar, but at times up to 6 h there appeared to be more recovery of DNA synthesis in resistant than in sensitive cells. Using the measured levels of DNA synthesis, different periods of pulse label were given 1 h after drug treatment in order to give similar levels of DNA synthesis in all samples, and the ligation of this newly synthesized DNA was followed using the hydroxyapatite technique. At equal doses, the labelled DNA appeared to be ligated more fully in resistant than sensitive cells, providing evidence for defective post-replication repair in sensitive cells.

### MEASUREMENT OF THE UPTAKE AND INTRACELLULAR LOCALIZATION OF DAUNORUBICIN USING FLOW MICROFLUORIMETRY

A. GARNER and B. W. FOX
*Paterson Laboratories, Christie Hospital and Holt Radium Institute, Manchester*

Uptake of daunorubicin (DnR, NSC-82151) by wild-type Yoshida cells in culture can be measured from the laser-excited emission of the drug in a flow system (Bio/Physics 4800A). The rate and extent of drug uptake increases with the concentration of the drug in the culture medium. At concentrations above 10 $\mu$M there is a build-up of cell debris due to the rapid and direct action of the drug, which may be distinguished from intact cells on the basis of particle size.

There is a large decrease in laser-excited emission from DnR-treated cells which have been fixed and treated with RNase. The emission is too weak to distinguish any cell-cycle pattern of staining, which might have been expected since DnR is known to intercalate in double-stranded DNA (Di Marco *et al.* (1976), *Cancer Res.*, **36,** 1962). The effects of incubation of cells with DnR and subsequent staining with acridine orange (AO) and ethidium bromide in flow microfluorimetry experiments was tested. The observations show that the binding of these drugs is affected by DnR, but in different ways which depend on the association constants of the dyes with DNA.

The ratio of red to green fluorescence from AO increases with the time of incubation of the cells with DnR, which indicates that DnR treatment of cells affects the ratio of double- and single-stranded DNA available for AO binding. This method was used to measure uptake of DnR into cell nucleoprotein, and the results were compared with DnR uptake measured by extraction into chloroform.

### MEASUREMENT OF MELPHALAN AND CHLORAMBUCIL AND THEIR METABOLITES BY A SINGLE HIGH-PRESSURE LIQUID-CHROMATOGRAPHY METHOD

A. G. Bosanquet, M. Henfrey and E. D. Gilby
*Department of Oncology, Royal United Hospital, Bath*

Measurements of melphalan (M) and chlorambucil (C) at the concentrations found in plasma during chemotherapy have recently become possible by high-pressure liquid chromatography (HPLC). However, either a gradient elution system has been necessary, or measurement of the degradation products of M (monohydroxymelphalan, MOH, and dihydroxymelphalan, $M(OH)_2$) is made difficult by their close proximity to the solvent front.

We have developed a method which uses an isocratic elution system suitable for the measurement of M, MOH and $M(OH)_2$ or C and its metabolite phenyl acetic mustard (PAcM). Sodium lauryl sulphate (SLS) is used in the mobile phase as an ion-pair reagent, and dansyl proline (DP) as an internal standard.

The drug plus added DP is first extracted from the plasma sample by washing through an XAD-2 column and eluting with methanol. This methanol eluate is injected directly on to the HPLC column and eluted with methanol/water containing SLS. It has been found that reduction of the methanol concentration or temperature causes an increase in all k′ values, whereas a pH change either way, or a reduction in SLS concentration, reduces the k′ of M, MOH and $M(OH)_2$ markedly and DP slightly, whereas the position of C and PAcM remain virtually unchanged.

### EFFECT OF LIPOSOME ENCAPSULATION OF ACTINOMYCIN-D ON ITS THERAPEUTIC EFFICACY

S. B. Kaye, J. A. Boden, K. D. Bagshawe and B. E. Ryman
*Departments of Medical Oncology and Biochemistry, Charing Cross Hospital, London*

Liposomes (phospholipid vesicles) are capable of prolonging the plasma clearance, modifying the tissue distribution and enhancing the *in vitro* activity of cytotoxic agents, and have therefore been proposed as drug carriers. We have compared the efficacy of actinomycin D (AD) encapsulated in small cationic liposomes with that of free drug, in a combined therapeutic-toxicity study. The model used was the Ridgway osteosarcoma (ROS) maintained by serial passage in AKR mice.

For free AD the minimum effective dose (MED—the dose which inhibited tumour growth to 10% of control tumour weight) was 0·3 mg/kg, and the $LD_{50}$ was 0·8 mg/kg. In contrast, with i.v. doses up to 8 mg/kg of AD encapsulated in liposomes, neither the MED nor the $LD_{50}$ have been reached. The maximum tumour-growth inhibition was 68% of control tumour weight, whilst survival of mice treated with 8 mg/kg of entrapped drug was 83%.

These results indicate that, despite a considerable delay in plasma clearance (the 5h plasma level of AD was 37-fold greater for entrapped than for free drug) the activity of AD encapsulated in liposomes in both host and tumour tissues is markedly reduced. This may partly be due to failure of drug uptake, since the peak ROS tumour AD concentration (expressed as percentage of injected dose per g of tumour at 24 h) was 6·0% $\pm$ 0·5 (mean $\pm$ s.e.) for free AD, and 2·0% $\pm$ 0·2 for entrapped AD. Further studies should determine whether liposome encapsulation of cytotoxic drugs may prove more useful for the treatment of drug-resistant tumours, or for those drugs whose activity is enhanced by delayed plasma clearance.

### EFFECT OF HYPERTHERMIA ON THE PHAGOCYTIC ACTIVITY OF TUMOUR-BEARING ANIMALS

S. A. Shah and J. A. Dickson
*Cancer Research Unit, University Department of Clinical Biochemistry, Royal Victoria Infirmary, Newcastle upon Tyne*

Previous work has shown that, following curative local heating of the rabbit VX2 carcinoma, tumour regression resulted in host cure, with an increase in both cellular and humoral immune competence (Shah & Dickson (1978) *Cancer Res.*, **38**, 3518). This work has been extended in the rabbit system and in a rat MC7 sarcoma model with a view to determining the role of macrophages in tumour regression following curative hyperthermia.

The phagocytic index (K) was determined at 7–10-day intervals by measuring the rate of clearance of colloidal carbon (8 mg/100 g body wt) from blood. Serum lysozyme levels, which have been claimed to reflect macrophage activity *in vivo*, were determined by a turbidometric method.

In untreated rabbits and rats, progressive tumour growth was accompanied by a 5-fold increase in K and a 3-fold increase in serum lysozyme levels. After curative heating of intramuscular VX2 tumours (15–20 ml, 47–50°C/30 min) and the MC7 sarcoma in the foot of rats (1–1·3 ml, 43°C/2 h), animals in which carbon clearance was sequentially tested showed a decreased tumour cure rate, from 70% to 17% in rabbits and from 100% to 50% in rats. Cured animals showed no decrease in elevated serum lysozyme. Tumour recurrence and death of the animals was associated with K and serum lysozyme levels comparable to those in untreated tumour-bearing animals. The lysozyme content of untreated MC7 sarcomas increased with increase in tumour mass, whereas the enzyme content of tumours decreased significantly over 7 days' curative heating.

The results suggest that K and serum lysozyme levels may be related to host tumour burden, but cannot be used to monitor tumour regression.

### NATURAL IMMUNITY IN RATS DEVELOPING SPONTANEOUS TUMOURS

G. R. Flannery and C. G. Brooks
*Cancer Research Campaign Laboratories, University of Nottingham*

A small proportion of animals in an inbred colony of WAB/Not rats spontaneously develop tumours in the breast or kidney. The spontaneous cytotoxicity of spleen cells from both tumour-bearing and age-, sex- and parity-matched control animals has been measured in a 6h release assay using target cells derived from a syngeneic methylcholanthrene-induced sarcoma. The natural cytotoxic activity of normal animals in the same age and parity range as the spontaneous breast tumour bearers has previously been shown to be comparable with that of young (8–12-week) animals, and to be mediated by non-phagocytic, non-adherent, non-T, non-B cells bearing Fc receptors. Spontaneous tumour-bearers exhibited levels of cytotoxicity occasionally lower than that of controls on a cell-for-cell basis, but at least as high as controls when total splenic lytic units were considered. Variations in reactivity were apparently unrelated to age, parity or tumour type. Thus, the development of spontaneous tumours in this system is not associated with either increased or decreased splenic natural immunity, a finding of considerable importance in view of the possible role or natural immunity in immune surveillance.

### BCG-INDUCED AUGMENTATION OF NATURAL CYTOTOXICITY IN THE RAT

M. R. Potter and M. Moore
*Department of Immunology, Paterson Laboratories, Christie Hospital and Holt Radium Institute, Manchester*

In the normal W/Not rat, natural (spontaneous) cytotoxicity in the peritoneal cavity is intermediate (3·4 lytic units/$10^6$ cells) between that of spleen (10·6 lu/$10^6$ cells) and lymph node ($<1$ lu/$10^6$ cells). I.p. injection of BCG (Glaxo) induced a significant dose- and time-dependent augmentation of cytotoxicity against the human erythroleukaemic cell line, K562, as determined by $^{51}$Cr release from pre-labelled target cells. Stimulation was maximal 4 days after rats were inoculated with 7 mg (dry wt) BCG, and heat-killed (1 h at 80°C) organisms were no less effective than viable preparations. A 5-fold increase in cytolytic activity was observed in BCG-induced exudates compared with the unstimulated population, determined at several E:T ratios. Under these conditions, activity in the peritoneal cavity (17·5 lu/$10^6$ cells) exceeded that of spleen cells (12·7 lu/$10^6$ cells) which, like lymphnode cells, showed little deviation from their respective levels in unstimulated hosts. Deployment of various fractionation procedures indicated that the augmented cytolysis was attributable to more than one cell type, but that a major component was similar to natural killer (NK) cells in untreated hosts. Augmentation of their activity appears to be a property of diverse agents which induce strong nonspecific immunity, and this effect may be linked to the induction or release of interferon. As such, these data could be relevant to current immunotherapeutic trials using these agents, including interferon itself.

### COMPARISON OF LYMPHOID CELLS INFILTRATING TUMOURS GROWING PROGRESSIVELY OR UNDERGOING IMMUNOTHERAPY - INDUCED REGRESSION

R. A. Robins, G. R. Flannery and R. W. Baldwin
*Cancer Research Campaign Laboratories, University of Nottingham*

Regression of a methylcholanthrene-induced transplanted rat sarcoma has been obtained after treatment with an inoculum of sarcoma cells in admixture with BCG. Previous studies have shown that this regression is accompanied by a marked lymphoid infiltration. Lymphoid cells have been separated by nylon-column filtration from both progressively growing and regressing tumours 10 days after inoculation, and these cells have been compared in short-term (6h) $^{57}$Cr-release, and long-term (60h) [$^{74}$Se] selenomethionine-uptake cytotoxicity assays, and for their capacity to control tumour growth *in vivo* in Winn-type assays. Although the yields of host cells from regressing tumours are greater than those from progressing tumours, no consistent quantitative or qualitative differences between the lymphoid cells from the two types of lesion have so far been detected in the *in vitro* or *in vivo* tests. These results argue against the generation of a new type of effector cell induced by the immunotherapy, and suggest that factors controlling extravasation and accumulation of lymphoid cells within tumours may be important in immunotherapy-induced tumour regression.

### NATURAL CYTOTOXICITY IN MAN: LOW SUSCEPTIBILITY OF FRESHLY ISOLATED TUMOUR CELLS TO LYSIS

B. M. VOSE and M. MOORE
*Department of Immunology, Paterson Laboratories, Christie Hospital and Holt Radium Institute, Manchester*

Blood lymphocytes from healthy donors and cancer patients have been tested for cytotoxic potential against freshly isolated lung tumour cells and the K562 cell line. While the majority showed high reactivity for the cell line, only very low levels of cytotoxicity could be detected against the tumour targets, except in 6/75 cases. In many individuals, cells were completely refractory under the conditions used (4h $^{51}$Cr release at an effector:target ratio of 50:1). This low susceptibility to lysis was confirmed in cold-inhibition assays, in which fresh tumour cells showed minimal capacity to interfere with killing of K562 targets. By contrast, K562 cells uniformly blocked cytotoxicity. These data would suggest that NK effectors can play only a minor role in protection against established human neoplasia. However, the susceptibility of freshly isolated tumour targets may not be so low as to be biologically irrelevant. By boosting levels of NK by treatment with interferon, and extension of the assay time to 18 h, higher levels of NK-mediated lysis could be detected.

### ANTIBODY INDUCTION BY 3-METHYLCHOLANTHRENE (MCA)-TREATED SPONTANEOUS TUMOUR CELLS AGAINST OTHER NON-MCA-INDUCED TUMOURS

J. G. REEVE and M. J. EMBLETON
*Cancer Research Campaign Laboratories, University of Nottingham*

It has previously been shown that rat embryo cells treated *in vitro* with MCA elicit antibodies in syngeneic rats which react specifically against certain established MCA-induced sarcomas, indicating the acquisition of new antigens cross-reacting with those sarcomas. We now report that antibodies can similarly be elicited by MCA-treated malignant cells, and that they may react against tumours not induced by MCA. Cells of a non-immunogenic spontaneous mammary carcinoma (Sp15) were treated for 18 h with MCA (10 $\mu$g/ml) in 0·5% acetone, or acetone alone, and washed $\times$ 6. Syngeneic rats were immunized with these cells and their sera were tested for membrane immunofluorescence against various types of established tumours. Sera from rats immunized against MCA-treated Sp15 cells were positive against an immunogenic spontaneous mammary carcinoma (Sp4) and an aminoazodye-induced hepatoma (D192A). Sera from acetone-treated Sp15 cells were negative, and this confirms the previously demonstrated inability of Tumour Sp15 to induce antibody responses in syngeneic rats. The results suggest: (a) that the induction of new antigenic specificities by MCA is not necessarily related to the process of malignant transformation, and (b) that the antigens themselves are not carcinogen-specific.

### ANTIGENIC CROSS-REACTIVITY BETWEEN BCG AND RAT TUMOUR CELLS

M. V. PIMM
*Cancer Research Campaign Laboratories, University of Nottingham*

The guinea-pig hepatoma Line-10 and human melanoma cells have been reported to cross-react antigenically with BCG (Borsos & Rapp (1973) *J. Natl Cancer Inst.*, **51,** 1085; Minden *et al.* (1974) *J. Natl Cancer Inst.*, **53,** 1325, and *J. Immunol.* (1976) **116,** 1407). In the present study the cross-reactivity of Line-10 and BCG has been confirmed, and a series of carcinogen-induced and spontaneous rat tumours has been examined for similar shared antigens.

Rabbit antiserum against BCG (DAKO Immunoglobulins, Copenhagen) reacted in an indirect-immunofluorescence test with BCG organisms (Glaxo) and with viable cells in suspension from Line-10 guinea-pig hepatoma, an aminoazo-dye-induced rat hepatoma (D23), an MCA-induced sarcoma (Mc106B) and a spontaneous mammary carcinoma (Sp22). Anti-Forsmann serum raised in rabbits against sheep red blood cells (Wellcome Reagents) also reacted with rat and guinea-pig tumour cells, but failed to react with BCG organisms, indicating that the component shared netween BCG and malignant cells is not classical Forsmann antigen. Absorption of anti-BCG serum with Line-10 cells abolished reactivity against both guinea-pig and rat tumour cells, and anti-BCG antibody adsorbed to and acid-eluted from Line-10 cells reacted with Line-10 cells and rat tumour cells, but not with normal rat cells, indicating common component(s) between Line-10, malignant rat cells and BCG.

This, and other reports of cross-reactivity between BCG and malignant cells, suggests that the immunotherapeutic effect of BCG may, at least in part, be due to their shared antigenic components.

### TUMOUR-SPECIFIC ANTIBODY PRODUCTION BY MOUSE MYELOMA-RAT SPLEEN CELL HYBRIDS

B. Gunn, M. J. Embleton and J. G. Middle
*Cancer Research Campaign Laboratories, University of Nottingham*

Hybrids between P3-NS1 mouse myeloma cells and spleen cells from rats immune to syngeneic tumours were prepared by fusion with polyethylene glycol. The tumours used were an aminoazo dye-induced hepatoma (D23) and a spontaneously arising mammary carcinoma (SP4), both of which are able to induce a specific antibody response in syngeneic WAB/Not rats. Supernatants from hybrid cells growing in selective (HAT) medium were tested for antibody activity against various tumour and normal cell targets, using an assay in which bound antibody was detected with $^{125}$I-labelled sheep F(ab)$_2$ anti-rat IgG. Antibody was detected in 1/38 supernatants from hybrids of P3-NS1 and Sp4-immune spleen cells, and in 2/76 supernatants from P3-NS1/D23-immune spleen cell hybrids. Specificity tests showed that these antibodies were specific for the tumour to which the spleen-cell donor was immunized. These results indicate the possibility of preparing monoclonal antibodies against tumour-specific antigens using syngeneic spleen-cell donors, thus avoiding many problems of specificity and the enforced rigorous selection associated with the use of xenogeneic spleen-cell-donors.

### PAPAIN SOLUBILIZATION OF RAT HEPATOMA D23 SPECIFIC ANTIGENS

M. E. Evans, D. Hannant, J. G. Bowen and M. R. Price
*Cancer Research Campaign Laboratories, University of Nottingham*

Tumour-specific antigens expressed upon the aminoazo dye-induced WAB/Not rat hepatoma D23 are integral plasma-membrane-associated glycoproteins, being sensitive to papain, which renders soluble antigenic glycopeptides reactive with concanavalin A, and of mol. wt 50,000–60,000. The release of surface components by papain has been studied in greater detail, using hepatoma cells metabolically labelled *in vitro* with $^3$H-fucose and $^3$H-leucine. With increasing concentrations of papain, under conditions which do not affect cell viability, the release of fucose- and leucine-labelled soluble materials followed similar kinetics, which were also comparable to the release/inactivation of WAB/Not alloantigens as determined by assaying residual antigenic activity on treated cells by a radioisotopic antiglobulin test. Fucosylated components displayed a higher mol. wt profile that leucine-labelled material and ~50% of these glycopeptides were retained on Sepharose 4B-con A columns, compared with less than 5% of leucine-labelled components.

These internally labelled surface components are being examined for the retention of tumour-specific antigens by their capacity to bind to specific immunoadsorbents containing immobilized syngeneic hyperimmune sera, and preliminary characterization of these columns using radioiodinated, papain-solubilized D23 antigen indicate that the immunoadsorbents contain specific antibodies predominantly of low affinity for soluble tumour antigens.

### LYMPHOCYTE RESPONSE TO PHA AND ITS RELEVANCE TO CANCER DETECTION

J. A. V. Pritchard, J. E. Seaman, I. J. Kerby, T. J. Deeley and B. H. Davies
*Immunology Department, Tenovus Laboratories, South Wales Radiotherapy and Oncology Service, Velindre Hospital, Cardiff, and Asthma Research Unit, Sully Hospital, S. Glam.*

Recent evidence (Pritchard *et al.* (1978) *Lancet*, ii, 1275) has shown that the successful operation of the SCM technique (Cercek & Cercek (1978) *Eur. J. Cancer*, **13**, 903) can be used to monitor lymphocyte response to PHA, and that this is dependent upon the sampling technique of the separated cell population. Lymphocytes separated from normal and benign-disease donors and sampled at the normal interface region (Band 1) of a modified Ficoll/Triosil gradient ($\rho = 1{\cdot}081$ g/cm$^3$ at 19°C) show characteristic responses to PHA when assessed by the SCM technique. Lymphocytes sampled at the same regions from subjects with malignant disease exhibit little if any response to PHA. However, response to PHA can be characteristically demonstrated for lymphocytes from patients with malignant disease when the region below the normal gradient interface is sampled (Band 2). A study of varying sizes of skin cancer has shown that a tumour of > 3 mm diameter is necessary for this phenomenon to occur in the lymphocyte population with a resultant PHA response in Band 2 cells. A study of benign and malignant chest conditions has shown that remarkable discrimination can be obtained between the groups.

### THE MODIFIED C1q TEST IN PATIENTS WITH BREAST CANCER

P. J. Doyle, R. A. Robins, P. J. Webb, R. W. Baldwin and R. W. Blamey
*Department of Surgery and CRC Laboratories, University of Nottingham*

A retrospective study using the C1q binding assay (Zubler *et al.* (1976) *J. Immunol.*, **116,** 232) was performed on heparinized plasma samples from patients with breast cancer. A relationship between the mean levels of binding for patients in different prognostic groups was demonstrated (Hoffken *et al.* (1978) *Lancet*, i, 672) but wide variation in results precluded prediction for individual patients. To standardize collection and storage, serum samples were chosen for a prospective study. Initial tests on serum did not distinguish between patients and controls. The addition of heparin to samples increased levels of binding in patients with breast cancer but had little effect on control samples. Preliminary results are:

| | Young adults | Benign breast disease | Breast cancer |
|---|---|---|---|
| Number | 12 | 25 | 39 |
| Mean ± s.d. | 4·82 ± 2·79 | 8·45 ± 5·03 | 18·12 ± 9·47 |

Pre-operative testing gave raised levels of binding in 31/39 patients with breast cancer, but only 6/25 patients with benign breast disease ($P < 0{\cdot}001$). In cancer patients with raised levels tested one month postoperatively, 80% of levels fell to normal. Cancer patients tested 6 months or more postoperatively were divided according to node status. The mean level for node-negative patients was 8·61 ± 4·65, for node-positive patients 17·70 ± 10·91.

These preliminary results suggest that the modified C1q binding assay is a useful marker in patients with breast cancer.

### *CORYNEBACTERIUM PARVUM* TREATMENT OF PATIENTS WITH BRONCHIAL CARCINOMA: A PROSPECTIVE CLINICAL TRIAL

H. D. Mitcheson and J. E. Castro
*Department of Surgery, Royal Postgraduate Medical School, Hammersmith Hospital, London*

62 patients entered a trial of *C. parvum* (CP) treatment for histologically confirmed bronchial carcinoma not amenable to operation. Patients were randomized to CP + radiotherapy or radiotherapy alone ("controls") with stratification for histological type, tumour size and spread.

*Patient entry:*

*Localized disease:* Squamous—CP,19; Control, 18. Anaplastic—CP, 2; Control, 2. Oat-cell —CP,1; Control, 2.

*Metastatic disease:* Squamous—CP, 11; Control, 4. Oat-cell—CP, 1; Control, 0.

*Refused treatment:* 2.

Mean age of patients receiving CP was 56·9 years and controls 58·3 years.

*Side effects:* CP (10 mg/m$^2$) given by i.v. infusion monthly to a total of 4 caused fever, malaise, rigors and pyrexia. Pyrexia lasted 6–72 h (mean 10 h) with a maximum mean temperature of 38·7°C. Systolic blood pressure dropped in most patients, and 3 patients needed treatment; 9 required antiemetics; 2 became confused and 8 had a flu-like illness. 4 patients refused to complete the course; 20 found the treatment easily acceptable. The remainder had an initial severe reaction but subsequent infusions had less effect.

*Preliminary overall patient survival* with localized disease is:

| 22 CP | | 22 Controls | |
|---|---|---|---|
| Alive | Dead | Alive | Dead |
| 10 | 12 | 10 | 12 |
| (14·0 ± 1·34) | (4·6 ± 0·9) | (12·3 ± 0·9) | (3·3 ± 0·7) |

(months ± s.d. from start of treatment)

We conclude that CP is safe and tolerated by most patients, but significant prolongation of survival has not yet been demonstrated.

### THE YORKSHIRE TRIAL OF IMMUNOPOTENTIATION WITH LEVAMISOLE IN SURGICAL LUNG CANCER: FOLLOW-UP OF 317 PATIENTS TO NOVEMBER 1978

H. M. Anthony, A. J. Mearns, M. K. Mason, K. Moghissi and D. A. Watson

*Department of Immunology, Leeds General Infirmary*

Patients undergoing operation for lung cancer at 3 centres were randomized to levamisole or placebo treatment. Treatment with 1 tablet (50 mg) 2–4 times a day (according to body wt) started 3 days before operation and continued 3 days a fortnight for 2 years.

Patients on levamisole showed a significant excess of deaths from cardiorespiratory failure in the 6 weeks after operation. These were attributed to cardiac autoimmune attack, fatal because of the patient's altered pulmonary circulation. Later follow-up of the patients will be reported in terms both of "all deaths" and of "cancer deaths" in order to detect any advantage which levamisole may give to those surviving early hazard. This report concerns follow-up of all trial patients to 1 year after the end of patient accrual.

### THE USE OF SENSITIZED PIG LYMPHNODE CELLS IN THE TREATMENT OF CARCINOMA OF THE OVARY

G. M. Turner and M. O. Symes
*Departments of Obstetrics and Gynaecology and Surgery, University of Bristol*

5 patients with carcinoma of the ovary, 4 at clinical Stage III and 1 at Stage IV, have been treated by injection of sensitized pig mesenteric lymphnode cells. In 3 patients the nodes were immunized against the patient's tumour and in 2 against her skin.

All patients had viable tumour present at the time of immunotherapy, in spite of the maximum possible surgery ± radiotherapy.

One patient died 12 days after treatment, and both a pelvic tumour mass and a hepatic secondary showed evidence of massive haemorrhagic necrosis. A further patient died 2 months after treatment, extensive pelvic and vaginal tumour deposits (present at the time of treatment) having completely disappeared. One patient had temporary relief from ascites formation lasting 2 months, and a further patient had a remission lasting 4 months, during which she lived a normal life and gained weight.

The fifth patient had a right pleural effusion associated with right pleural tumour deposits. She received pig cells by intrapleural and i.v. injection, and is alive and free from tumour recurrence 4 years and 8 months later.

**(Abstracts of the BASO members papers are appearing separately in Chemical Oncology)**